# Retraction: Genome-wide characterization of Src Homology-3 (SH3) domain-containing proteins in the development and pathogenicity of *Fusarium graminearum*

**DOI:** 10.1371/journal.ppat.1014445

**Published:** 2026-07-20

**Authors:** The PLOS Pathogens Editors

Following the publication of the uncorrected proof of this article [1] the authors contacted PLOS to correct an inadvertent panel duplication affecting the panels ∆*Fgsh3B* and ∆*Fgsh3C* of Fig 3A. During the editorial assessment, the editorial team also identified repetitive areas within panels ∆*Fgnpb2* and ∆*Fgbbc1* of Fig 3A.

The corresponding author YL stated that panel Δ*Fgsh3B* was erroneously duplicated and presented as Δ*Fgsh3C* and that errors were made during the figure preparation of the Fig 3A panels ∆*Fgnpb2* and ∆*Fgbbc1*. They provided an updated version of Fig 3 as well as the underlying original images, but these data did not resolve the concerns regarding panels ∆*Fgnpb2* and ∆*Fgbbc1*. In addition, during editorial assessment of these images, PLOS identified partial overlap between panels representing different conditions.

In light of the above unresolved concerns that question the reliability and integrity of these data, the *PLOS Pathogens* Editors retract this article [1].

SJ, QC, HZ, WZ, and YL agreed with the retraction. YSA and ZW responded but expressed neither agreement nor disagreement with the editorial decision.
